# Cytological Features of a Metastatic Angiosarcoma in the Lymph Node Diagnosed via Liquid-Based Cytology

**DOI:** 10.3390/diagnostics13122124

**Published:** 2023-06-20

**Authors:** Jie-Yang Jhuang, Chih-Yi Liu, Min-Hui Tseng, Shih-Sung Chuang

**Affiliations:** 1 Department of Pathology, Mackay Memorial Hospital, New Taipei City 251, Taiwan; 2 Division of Pathology, Sijhih Cathay General Hospital, New Taipei City 221, Taiwan; 3 School of Medicine, College of Medicine, Fu Jen Catholic University, New Taipei City 221, Taiwan; 4 Department of Pathology, Chi-Mei Medical Center, Tainan 710, Taiwan

**Keywords:** angiosarcoma, cytologic features, fine needle aspiration, liquid-based cytology, vasoformative features

## Abstract

Angiosarcoma is a soft tissue sarcoma of vascular origin, with more than half of the cases arising in the skin and affecting primarily the face and scalp of elderly males. Furthermore, cutaneous angiosarcoma exhibits a higher incidence of lymph node metastases than other types of sarcomas. Angiosarcomas are rarely aspirated and are occasionally encountered on cytological samples. It is a diagnostic challenge in evaluating fine needle aspiration (FNA) from a metastatic angiosarcoma without the knowledge of prior history. We present a case of scalp angiosarcoma with disease progression to erythroderma and cervical lymphadenopathy 20 months after. FNA of the cervical node revealed vasoformative features, including hemophagocytosis, formation of an intracytoplasmic lumen/vacuole, endothelial wrapping, and cell grasping. The diagnosis of nodal metastasis by angiosarcoma was confirmed with immunohistochemistry (IHC) using two vascular markers on cell block sections. Our case demonstrates the recognizable cytomorphologic clues for this rare metastatic malignancy.

Angiosarcoma is a soft tissue sarcoma of vascular origin with aggressive clinical behaviors, and accounts for approximately 2–4% of soft tissue sarcomas [1]. More than 50% of cases arise from the skin, with the remainder occurring within deep soft tissues, breast, bone, or viscera [1]. Cutaneous angiosarcoma primarily affects the face and scalp of elderly males [2], and is characterized by a locally aggressive course, difficulty to be completely excised, and a poor prognosis due to the high potential for metastasis [3]. Furthermore, there is a higher chance for lymph node metastasis occurrence compared to other types of sarcomas [4,5,6]. Metastatic angiosarcoma is rarely encountered in fine needle aspiration (FNA) samples that can closely mimic other malignancies. There is a wide cytological spectrum, ranging from a predominance of epithelioid cells in some cases, to a predominance of pleomorphic cells in others.

We present a case of scalp angiosarcoma with cervical lymphadenopathy. FNA liquid-based cytology (LBC) showed several vasoformative features of the tumor cells. We subsequently confirmed the diagnosis of nodal metastasis from angiosarcoma with a limited panel of immunohistochemical markers using cell block sections.

The clinical presentation of this patient, including red skin (localized erythroderma) and swelling over the head and neck area raises the clinical suspicion of erysipelas, cellulitis, inflammatory dermatitis (such as psoriasis and eczematous dermatitis), lymphatic/vascular metastasis of carcinoma, and cutaneous T-cell lymphoma (such as mycosis fungoides/Sézary syndrome) (Figure 1). When there is concomitant regional nodal enlargement, lymph node aspiration cytology becomes a helpful diagnostic approach.

In the current case, FNA cytology revealed atypical epithelioid cells with cytomorphology deemed to be unusual for a metastatic carcinoma (Figure 2). Ancillary studies are needed to determine the tumor type and the possible tumor origin. In biopsy samples, we can easily perform a broad panel of immunohistochemistry for a wide range of differential diagnoses. However, cytology specimens, including cell block sections might be limited as it was for our case. By reviewing the patient’s past history, we found that he had cutaneous epithelioid angiosarcoma diagnosed 20 months earlier and received local radiotherapy alone. The current clinical manifestation of erythroderma and cervical lymphadenopathy indicated disease progression with nodal metastasis, which was confirmed using FNA cytology with a limited panel of immunohistochemistry with cell block sections (Figure 3).

Lymph node metastasis is uncommon in soft tissue sarcomas. The reported incidence of lymph node metastasis in the literature varies widely. In a recent study on the surveillance, epidemiology, and end Results (SEER) database, the overall lymph node metastasis rate was 6.02% [6]. The top three most common subtypes exhibiting lymph node metastases were rhabdomyosarcoma (26.88%), angiosarcoma (15.43%), and sarcoma NOS (9.39%), respectively [6]. Keung et al. reported an incidence rate of 3.5% among all the sarcoma patients from the National Cancer Data Base [5]. They also found that angiosarcoma (6%), epithelioid (13%), clear cell (16%), and small cell sarcoma (19%) had the highest incidence of lymph node metastasis [5]. The incidence of lymph node metastasis from angiosarcoma has been reported to be less than 15% (6% and 13.5%) in two large case series [5,7]. However, recently, Kang Y. et al. reported that 52.5% of their 40 cases of scalp angiosarcoma showed regional nodal metastasis, an incidence that is much higher than the other types of sarcoma [4]. Accordingly, they suggested that for patients with scalp angiosarcoma, the initial curative surgery should include the prophylactic evaluation of regional lymph nodes for pathologic nodal staging, prognosis estimation, and the decision for systemic treatment [4].

FNA cytology is an invaluable diagnostic modality to diagnose metastases, even for metastatic sarcoma. The diagnostic yield of lymph node FNA for metastatic sarcoma has been rarely documented. The concordance rate between cytological and histological diagnosis reached 89.5% and 82.6%, respectively, based on the two studies on FNA of metastatic sarcoma [8,9]. However, metastatic sarcoma has rarely been encountered on FNA samples. Lymph node aspirates should therefore be carefully searched for atypical cells, particularly in paucicellular smears as in our case.

Angiosarcoma is a rare neoplasm, with less than a 100 cases having been reported on FNA cytology in the English literature. Geller et al. reviewed the cytologic features of 26 cases of angiosarcoma diagnosed on FNA in conventional smears [10]. They found that abnormal mitoses were the most frequent (85%), followed by single malignant cells (81%, including epithelioid [69%], spindled [62%], and plasmacytoid [19%] cells, respectively). The latter feature makes poorly differentiated carcinoma a diagnostic pitfall. In addition, three-dimensional clusters were found to be common (54%). The most distinctive finding was vasoformative features, which were identified in 88% of cases with at least one of the following: hemophagocytosis (54%), cytoplasmic lumina/vacuoles (69%; containing red blood cells [54%] or neutrophils [31%]), and endothelial wrapping (69%). They considered these features to be important diagnostic clues for angiosarcoma [10]. In contrast to conventional preparation, Jung and Kim reported a metastatic angiosarcoma diagnosed from liquid-based cytology [11]. The following cytological features different from conventional preparation were emphasized: a clean background, absence of hyperchromatic nuclei, intracytoplasmic vacuoles with peculiar shapes, juxtanuclear condensation, and perinuclear clearing [11]. Similarly, in our case, the LBC preparation with SurePath made it easier to identify hemophagocytosis, endothelial wrapping, and cytoplasmic lumina/vacuoles without the bloody background in conventional preparation. Different preparation methods may affect the background and cytomorphological details. In our opinion, standardized fixation and preparation in LBC may provide better, fixed tumor cells and clean background compared to the conventional preparation for cytological evaluation and make it easier for identifying the vasoformative features.

Diagnosing angiosarcoma by FNA cytology is challenging. Our case illustrates the vasoformative features and history of prior angiosarcoma, with the aid of immunohistochemical markers contributing toward a correct and timely diagnosis.

## Figures and Tables

**Figure 1:**
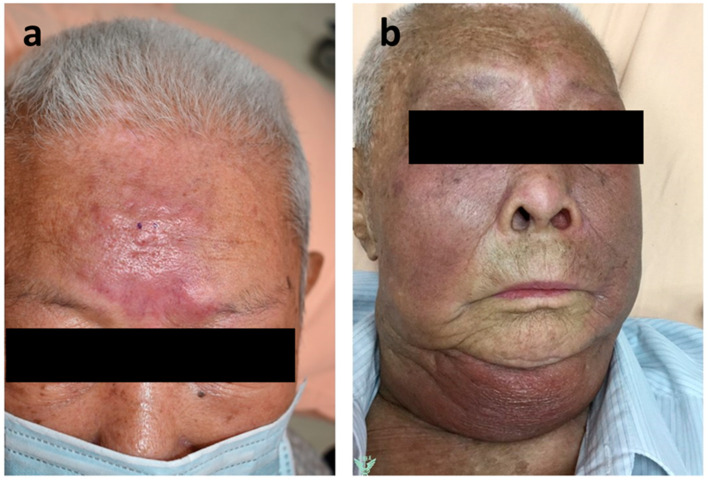
An 88-year-old Taiwanese male initially presented with ill-defined, erythematous patches over the forehead in November 2020. (**a**) Twenty months later, he developed confluent, erythematous patches from the head to the neck in September 2022. (**b**) Neck CT scans revealed diffuse, cutaneous, and subcutaneous infiltrations in the head and neck areas, being more prominent on the left side, with the imaging differential diagnoses of cellulitis, metastasis, among others. Subsequent neck sonography showed enlarged lymph nodes in the bilateral neck regions, up to 2.3 cm in size.

**Figure 2:**
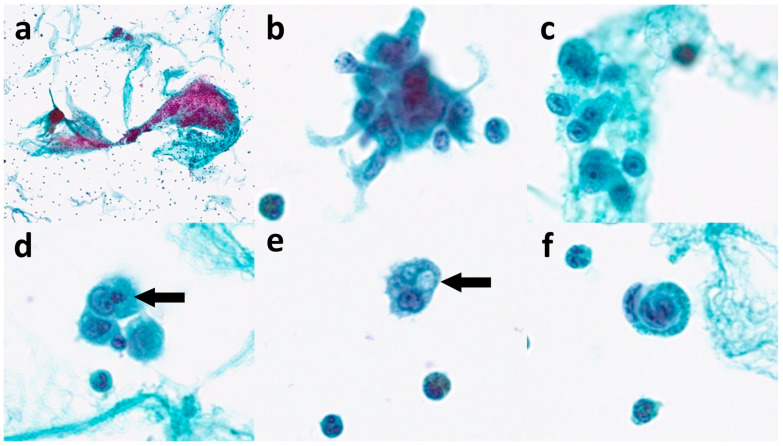
Sono-guided FNA cytology of the left cervical lymph node was performed smoothly and prepared into a SurePath slide (Becton, Dickinson and Company, Sparks, MD, USA). The nodal FNA cytology specimen showed a low cellularity, comprising singly scattered, and loosely or tightly clustered tumor cells with three-dimensional clusters arranged similarly to streams of fishes in the fibrous stroma of the microtissue ((**a**) Papanicolaou stain, ×40). The tumor cells exhibited a variable appearance, from spindled to epithelioid or plasmacytoid with abundant cytoplasm ((**b**,**c**), Papanicolaou stain, ×800). The tumor cells were large in size and highly atypical with round-to-oval nuclei and prominent nucleoli observed ((**c**), Papanicolaou stain, ×800). There were occasionally vasoformative features observed, including the hemophagocytosis of neutrophils (arrow) or RBCs ((**d**), Papanicolaou stain, ×800), intracytoplasmic lumen (arrow) ((**e**), Papanicolaou stain, ×800), and cells-in-cells or cell grasping ((**f**), Papanicolaou stain, ×800). The differential diagnoses based purely on the cytological findings of epithelioid cells were broad, and might include poorly differentiated carcinoma, sarcoma, and less likely, large cell lymphoma and melanoma. However, the vasoformative features, including hemophagocytosis, intraluminal vacuole/lumina, and endothelial wrapping pointed toward the possibility of malignant vascular tumor, or angiosarcoma. Under the suspicion of angiosarcoma, the past history of this patient was reviewed. In November 2020, the initial presentation was noted as ill-defined, erythematous patches over the forehead. Incisional biopsy of the skin lesion was undertaken and diagnosed with cutaneous epithelioid angiosarcoma. After the pathological diagnosis of angiosarcoma, he received radiation therapy for the scalp lesion.

**Figure 3:**
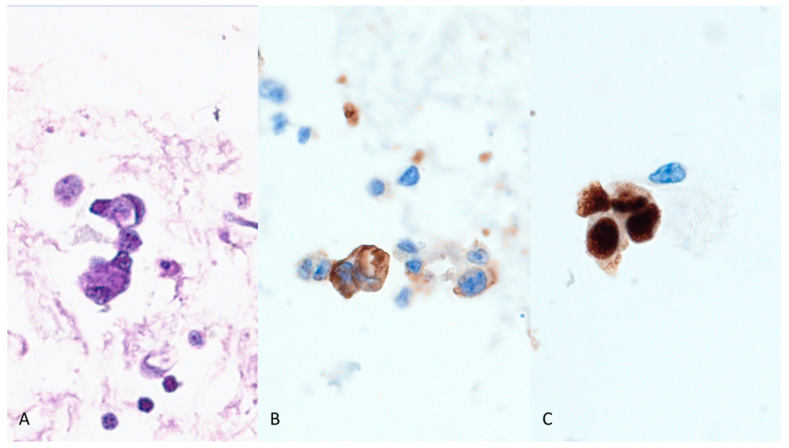
We prepared a cell block from the FNA specimen. The tumor cells, although small in number, were large in size and highly atypical with intra-cytoplasmic lumen formation ((**A**), H&E stain, ×800). With the presentation of metastatic malignancy suggestive of angiosarcoma, we performed only two immunohistochemical stains using cell block sections with the endothelial cell markers CD31 ((**B**), ×1000) and ERG ((**C**), ×1000). The tumor cells were found to be positive for both markers, confirming the diagnosis of angiosarcoma.

## Data Availability

Data are available on request due to all institutional restrictions related to patient privacy.
